# Supplementary material for: A distal enhancer guides the negative selection of toxic glycoalkaloids during tomato domestication
Source: Nat Commun. 2024 Apr 3;15:2894. doi: 10.1038/s41467-024-47292-7 (PMC10991328; doi:10.1038/s41467-024-47292-7)
Supplement: Supplementary file 1 — Supplementary Information [file 41467_2024_47292_MOESM1_ESM.pdf]

Supplementary Information for

# A distal enhancer guides the negative selection of toxic glycoalkaloids during tomato domestication

Feng Bai[1,7], Peng Shu[1,2,7], Heng Deng[1], Yi Wu[1], Yao Chen[1], Mengbo Wu[1], Tao Ma[1], Yang Zhang[1], Julien Pirrello[3], Zhengguo Li[4], Yiguo Hong[5,6], Mondher Bouzayen[1,3]*, Mingchun Liu[1]*

[1]Key Laboratory of Bio-Resource and Eco-Environment of Ministry of Education, College of Life Sciences, Sichuan University, Chengdu, 610065, Sichuan, China

[2]Clinical Medical Research Center, Xinqiao Hospital, Army Medical University, Chongqing 400037, China

[3]Laboratoire de Recherche en Sciences Végétales-Génomique et Biotechnologie des Fruits-UMR5546, Université de Toulouse, CNRS, UPS, Toulouse-INP, Toulouse, France

[4]Key Laboratory of Plant Hormones and Development Regulation of Chongqing, School of Life Sciences, Chongqing University, Chongqing, China

[5]School of Life Sciences, University of Warwick, Warwick CV4 7AL, UK

[6]State Key Laboratory of North China Crop Improvement and Regulation and College of Horticulture, Hebei Agricultural University, Baoding 071000, China

[7]These authors contributed equally: Feng Bai, Peng Shu

*Corresponding author. Email: mcliu@scu.edu.cn; mondher.bouzayen@toulouse-inp.fr

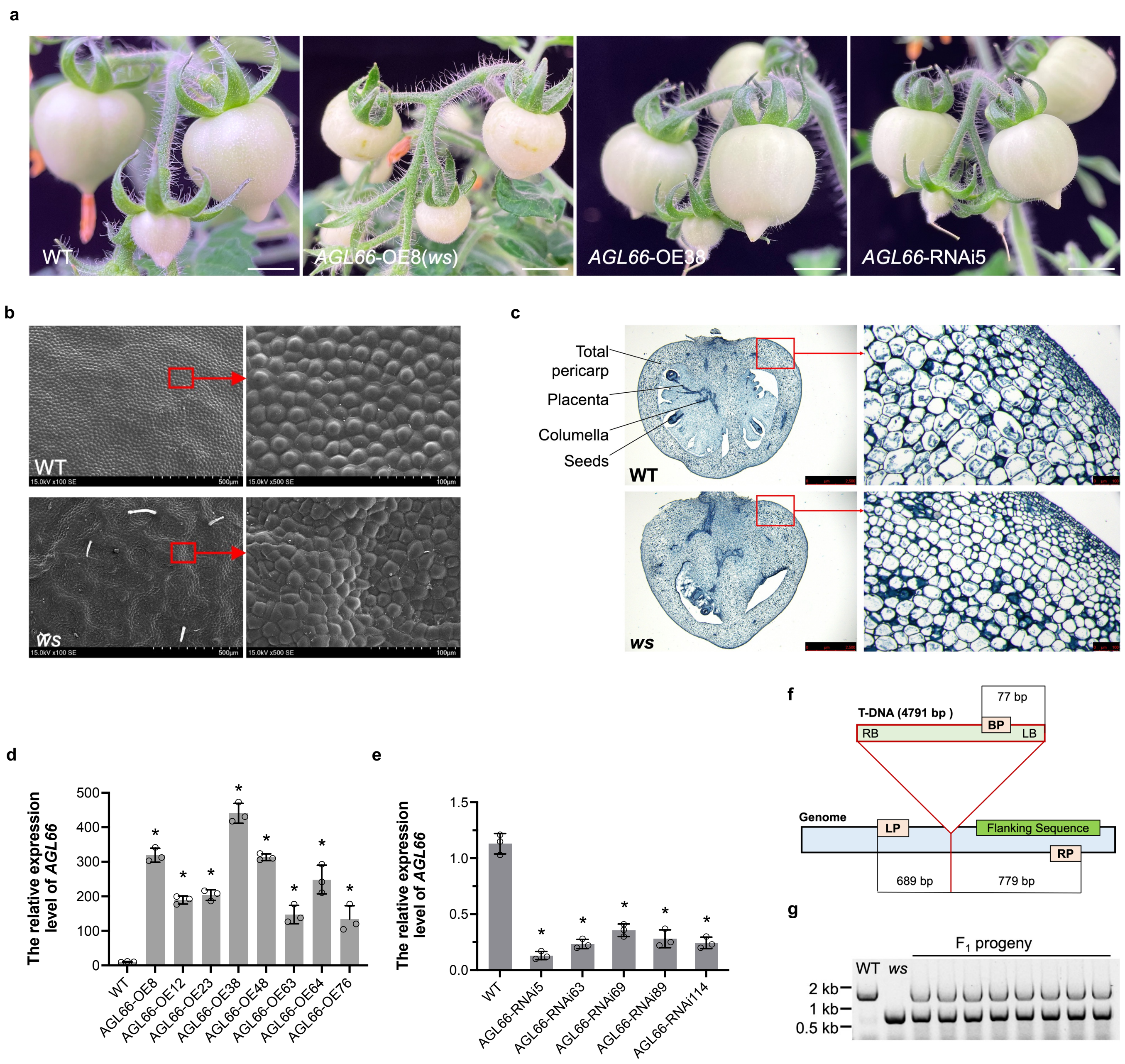


**Supplementary Fig. 1. The wrinkled surface of *ws* mutant fruits.**

**a**, Fruits of WT, *ws* mutant, and one *AGL66* overexpression (OE) line (*AGL66*-OE38), and one *AGL66* downregulation line by RNAi (*AGL66*-RNAi5) at 17 DPA. Bar = 10 mm. Line *AGL66*-OE38 shows the highest up-regulation of *AGL66* among all OE lines, and line *AGL66*-RNAi5 shows the highest down-regulation among all RNAi lines. These results suggest that the fruit wrinkled surface phenotype of *AGL66*-OE8 (wrinkled surface mutant, *ws*) comes from the T-DNA insertion, and not the altered gene expression of *AGL66*. **b,** SEM micrograph of surface at 7 DPA fruits. Bar=500 μm (left) and 100 μm (right). The picture is representative of at least 5 independent fruits of mutant all showing similar results. **c**, Microscopic cross-sections of 7 DPA fruits stained with safranin-fixed green. Bar=2.5 mm (left) and 100 μm (right). The picture is representative of at least 5 independent fruits of mutant all showing similar results. **d, e,** RT-qPCR analysis showing the relative expression of *AGL66* in *AGL66* overexpression lines (**d**) and *AGL66* downregulation lines by RNAi (**e**). Data are presented as mean ± SD (n=3). *$P < 0.05$ in one-way ANOVA . **f,** Schematic illustration of the primers used in the PCR assays. LP, Left genomic primer. RP, Right genomic primer. BP, T-DNA border primer. **g**, Identification of T-DNA insertion by PCR analysis.

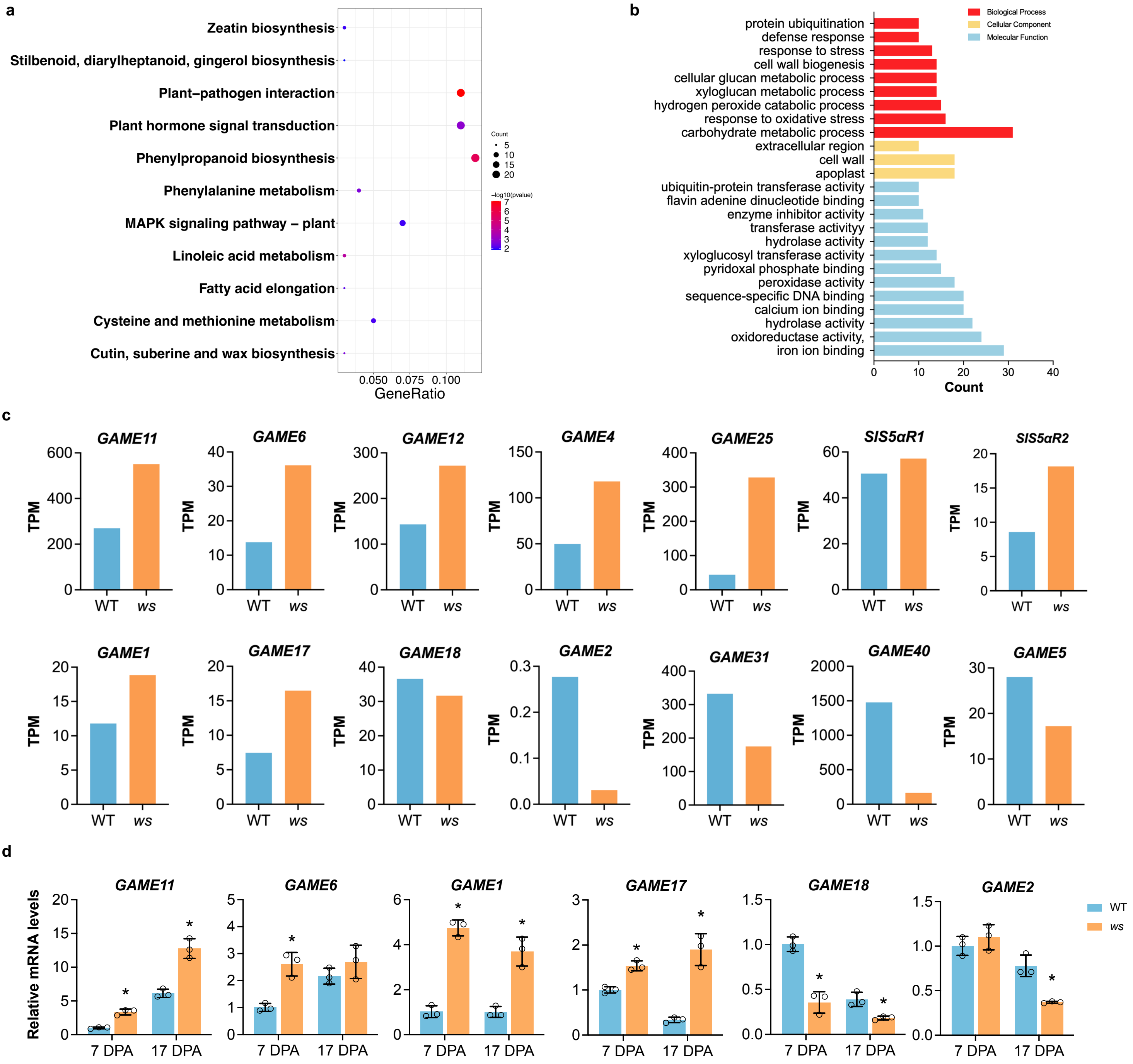


**Supplementary Fig. 2. RNA-seq analysis of WT and *ws* mutant fruits.**

**a**, KEGG pathway analysis based on the RNA-seq dataset of WT and ws fruits at 7 DPA. **b**, GO analysis based on the RNA-seq dataset of WT and ws fruits at 7 DPA. **c**, Comparison of SGAs biosynthesis genes expression patterns obtained by RNA-seq in WT and *ws* fruits at 7 DPA. **d**, RT-qPCR analysis of the relative expression of six *GAME* genes clustered on chr07 at 7- and 17-DPA. Data are presented as mean ± SD (n=3). **P* < 0.05 in multiple unpaired tests using Benjamini–Hochberg methods.

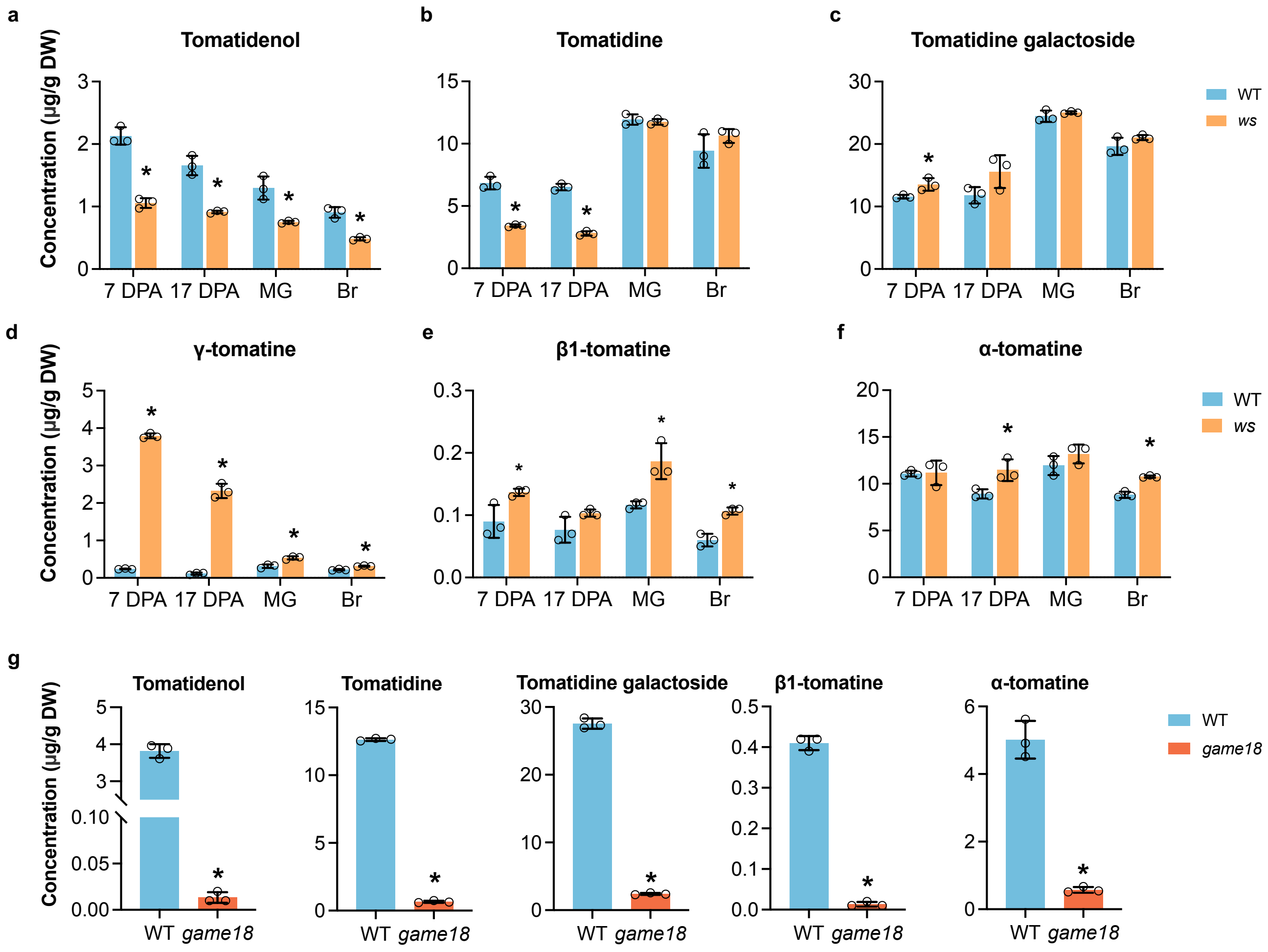


**Supplementary Fig. 3. *ws* mutant displayed altered SGAs profile.**

**a-f**, SGAs profiles of WT and *ws* fruits during ripening (7 DPA, 17 DPA, MG, and Br stages). DPA, Days post anthesis. MG, mature green. Br, breaker. UPLC-MS was used for SGAs profiling. Data are presented as mean ± SD (n=3). *$P$ < 0.05 in multiple unpaired tests using Benjamini–Hochberg methods. **g**, SGAs profiles of WT and *game18* fruits at 17 DPA. The value is presented as the mean ± SD (n=3) . *$P$ < 0.05 in two-sided ***t*-**test**.**

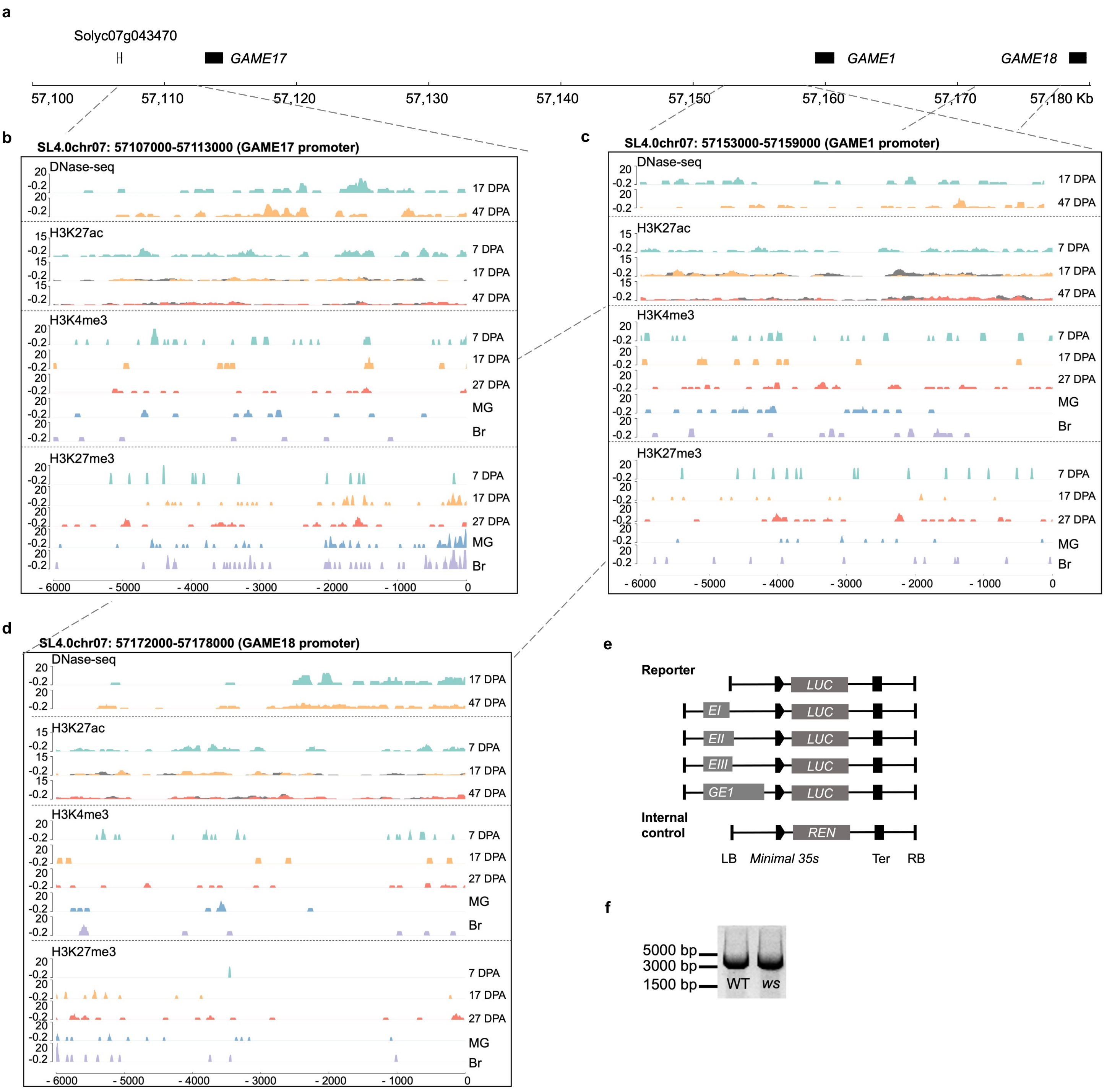


**Supplementary Fig. 4. The DHS and epigenomic features of promoters of three *GAME* genes near the T-DNA insertion site.**

**a,** The schematic representation of three *GAME* genes. DHS and histone modifications (H3K27ac, H3K4me3 and H3K27me3) profiles in the 6 Kb region of the promoter of *GAME17* (**b**), *GAME1* (**c**), and *GAME18* (**d**), visualized by pyGenomeTracks (PGT). Active enhancers can be identified by features such as high levels of chromatin accessibility, H3K4me3 and H3K27ac, and low levels of H3K27me3. **e,** Schematic illustration of the reporter and effector plasmid used in the transient assay. **f,** PCR analysis shows that *GE1* can be amplified by using cDNA as template.

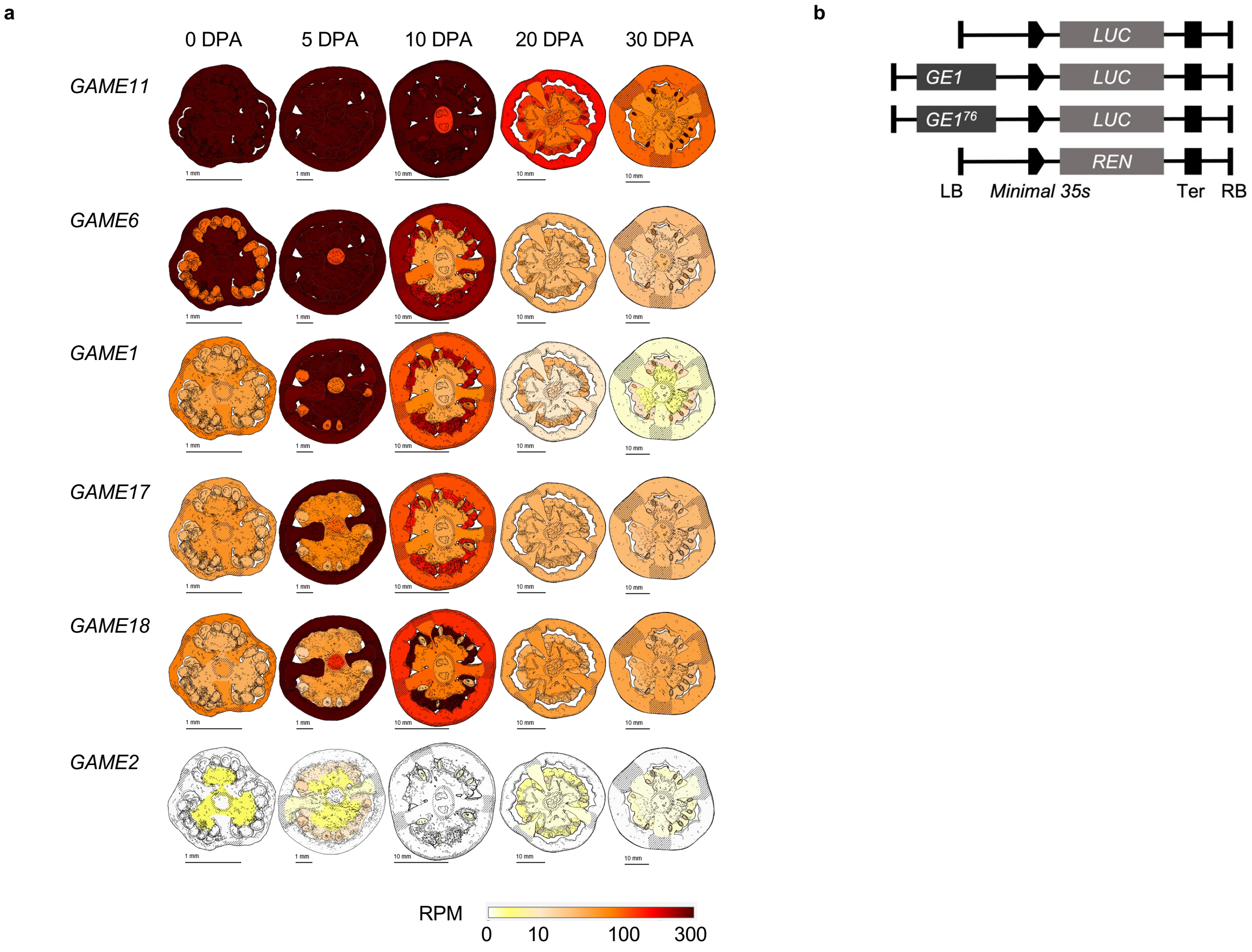


**Supplementary Fig. 5. Expression data of the six *GAME* genes.**
**a,** Tissue-based expression images of *GAME11, GAME6, GAME1, GAME17, GAME18* and *GAME2* genes. Data were extracted from the Tomato Expression Atlas platform (https://tea.solgenomics.net/). RPM, reads per million mapped reads. **b,** Schematic illustration of the reporter and effector plasmid used in the transient assay.

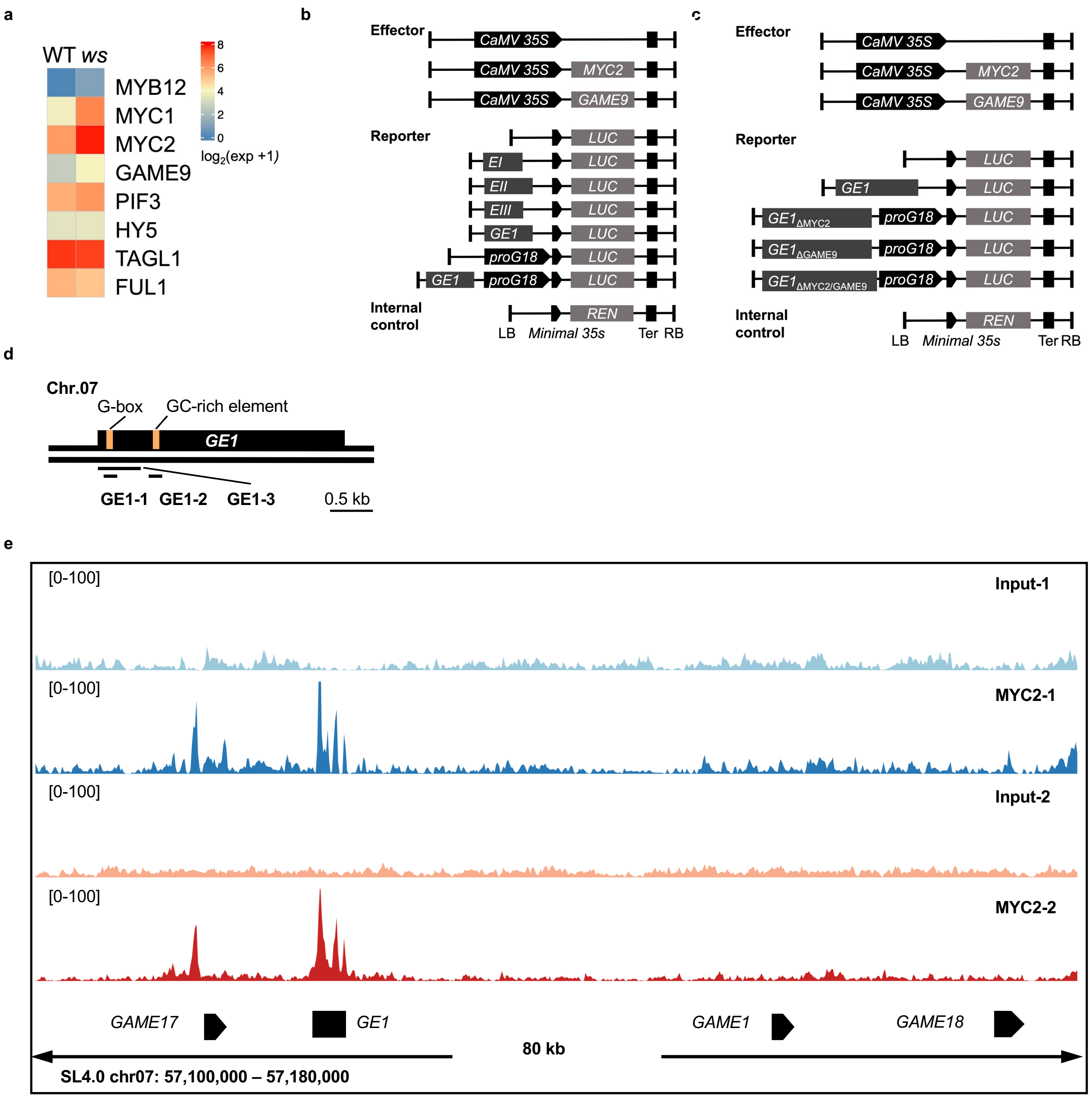


**Supplementary Fig. 6. GAME9 exerts its activity by cooperating with MYC2.**
**a,** Heatmap of genes involved in the regulation of SGAs biosynthesis in WT and *ws* fruits based on RNA- seq datasets. **b,** Schematic illustration of the reporter and effector plasmids used in the transient assay. **c,** Schematic illustration of the effector and reporter constructs used in the transient assay. **d,** Schematic diagram of GE1-1, GE1-2 and GE1-3 positions used in EMSA and DNA Pull-down assays. **e,** The ChIP-seq analysis of MYC2 showing the fold enrichment of MYC2 at the promoter of *GAME17* and the region of *GE1*. There was a stronger signal of MYC2 on *GE1* than that of *GAME17* promoter.
