# Supplementary material for: A distal enhancer guides the negative selection of toxic glycoalkaloids during tomato domestication
Source: Nat Commun. 2024 Apr 3;15:2894. doi: 10.1038/s41467-024-47292-7 (PMC10991328; doi:10.1038/s41467-024-47292-7)
Supplement: Supplementary file 3 — Description of additional supplementary files [file 41467_2024_47292_MOESM3_ESM.pdf]

DESCRIPTION OF ADDITIONAL SUPPLEMENTARY FILES DOCUMENT

Supplementary Data 1. Whole genome sequencing and analysis of the WT and ws mutant lines.

Supplementary Data 2. RNA-seq analysis of WT and ws mutant lines.

Supplementary Data 3. Kyoto Encyclopedia of Genes and Genomes (KEGG) pathway enrichment analysis of differentially expression genes (DEGs) between WT and ws.

Supplementary Data 4. Gene Ontology (GO) term enrichment analysis of DEGs between WT and ws.

Supplementary Data 5. SGAs profiles of WT and ws fruits during ripening.

Supplementary Data 6. Genome-wide interaction matrices showing chromatin loops in WT and ws at 7 DPA obtained by analyzing Hi-C sequencing data.

Supplementary Data 7. The 46 tomato accessions used for GE1 alignment.

Supplementary Data 8. Alignment of GE1 sequences used for the generation of the phylogenetic tree.

Supplementary Data 9. SGAs profiling of 22 tomato accessions.

Supplementary Data 10. The expression of genes shown to be involved in the regulation of SGAs biosynthesis extracted from RNA-seq profiling data.

Supplementary Data 11. All primers used in this study.

Supplementary Data 12. Gene and promoter sequence used in this study.
