# Supplementary material for: A distal enhancer guides the negative selection of toxic glycoalkaloids during tomato domestication
Source: Nat Commun. 2024 Apr 3;15:2894. doi: 10.1038/s41467-024-47292-7 (PMC10991328; doi:10.1038/s41467-024-47292-7)
Supplement: Supplementary file 15 — Supplementary Data 12 [file 41467_2024_47292_MOESM15_ESM.docx]

**Supplementary Data 12. Gene and promoter sequence used in this study.**

**GE1** (SL4.0chr07, 57,123,127-57,126,049 2922 bp, *Solanum* *lycopersicum* *cv* MicroTom)

The sequence in blue font is the EI fragment, and in yellow font is the EII fragment, and in red font is the EIII fragment. The sequence highlighted in yellow is the G-box motif and highlighted in green is the GCC-box motif.

AAATAAACATGTGCAAAATTCGTTTAAATTATTGTGAGGTGCTATAGATATGAAAAATCTTTATAACTAGAAAATTTAATAAGAATTGGGTCGGAATAATATTTTATTTATACTATTTTAGAATGATATTTTATTCATATTATTTTATTATTTTAAATATTTTTATCTTTTTAATTAAAATGGAAAAAAGATTAATTTATTTTAAAATAAAATAAATATTATAATTTTTTAATTTTCTAATCTGATTAAATTTTTTCATCGTTTAGCAATATACAAAAATATAATGTAGCTGTATTTTAAAAAGAAAAAAATCATTTTGACCAAAATTTAGCTAGAATGTATTTTATCCATATTATTTTTTACCTTTTTAAATATTTTTATTACTTTAACTTTCATGTTTTTAACTAAAAGTTCAAAAAAAATATTTTAATATAAAAGCTAGAATGATTTAATCGATATTATTTTTTCAATATTTTTATTATTTTAACTTTCATATTGTTTAACTAAAAATAAATAAAAAAAAAGATAAATTTATTTAATATAAAAAGAAATATAATAGACTTTTTAAAGTTTTGTTCTGGCTGGCCAGCCGTGGAACATAAATAAACGTGTGTAAAAATTCGTTGAAATTATTGTGAGGTGCTATAGATATGCACAGTTATTGTTATTAATAATTAATTATTGATGATCATAACCAACGACAAAAGGAAGTACATGTCATTTTTCTACATTTTGGTTTCTTTCTGGTAATTTAAATTTAGGAATGGGTTAAATAATTAGAAAATTTGGTTAGTATTTGGTTATGAAATTAAAATAATTATAATATTTTTTAATTTTAAAATAAATTGATTTTTTATTTAATTTTTTATTTAAAAGATAAGAATAAAAATATTTTAAAAGATAAAATAGCATAGAAAAATTATGATCTGATCAAAAAAAAAAATCCCTTTAAGTTAATAACTTCGATAATTAAATACTCTCACTATTTTTGGAGTTTTTTTATTATTTAAAATAATTAAATTAGTTAAGTTCATGATGAATATTTGATTTATTTATATTTATCCTTTTCTTTTTGATGAATTTTTATATCTTTTTAGAGATAAATTGCATTATTTTTAAAATTATATTTATAATTTTAAAATATTATTAAAATATTTACTTAACTATTTTCATTACAAATATATTGGATTATCTCACAAATGCAAATATATAGTATAAAAAGGACTGAAGGAGTGTTAACGAACGTACATCGCCATAACACTTATGTAAACAAAAAATCAGAACAAAGTTACAACACTTACTTCGAAGTTCTATATATTTATATATATATATATTATATTAGAAAGGAGTTTCAAGTCTTTTTCAATATGTCAGATTTCTGACATTAAATTAAAGTTTTGGTGGTATTAAGGCCAACTTCTAGAAATTAATATTTCATAAATTAATAATTTTTTTAAAATAATATTTTTTCTGATCATAATTTAGGCTAATAGAAAAAAGATCATCAATTTTGATTACATGATATATTTCAGAAGACCCTATATAAATATATTGTCCCATTAATATCATAAATTAATAATTCTTTAAAGGTACAAGCATACTTAAGATAATTTAGTGAAATATGATTAGATTATGTTCTGTTTTTTCTTAAAATTTAAATTTGTGAAGAGTTCTAGATACGATTAGGGTTCCTTATGTGGTTCCTCTAAACATGTATCACTTTCACCCGGATAATTCACCAGATTTTTGATATCGATTTTATTGTCGTAATCGAAATCATTAATCATAATCTCAAGTTAAACTCGTAGATAATTGAGAGTCTATAATTAAATTAATTAGTAGTGTATGCACATTTTCTTTATTTTTATCCTAATTGTTATTTTAACAATAACATATTAATAATAAATTTTAAAATATAATTAATAAAATTATTCCGTAAAATGTTTTTGAAAAAAAAATTAAAAATAAGAACCTTAATGACTATTAAAATAAATGTATAATCTTTCGCTAATATATAAATAGTAATACTTTATATGTGCATGAAGTGTCGCTTTAAGTATGTAATGGTATAATATTTCCATGAGTAAAATAATTTATTATTTGAAAATCAACATGTTCATGGATCCTGAGCGTATTCAATTTTTCCTATGCAATACTATGCGTGATTAATAATGATGCGTTTGTTAAGATTAAAAATATTACTTTTAGAAATAATTTCTAGGAAAATAAATATGTTTCTTTTTTCTTTTGATAGGTTGACAAAAAGTATTATCTTAAAATATTTTTATATAATTTAAATAATTATCACGTGTAAATTTTTTCATATGTCAATTATAAAAAATATTTTATTATTTTAAAAGAAAAAAATTATTTCCTAAAGAAAATATATTGTAAAAAATTTAACTGATTAAATATAGAAAAATTAACATTCTACATAATGAACACCACCTTAAAATTATTAAATTTCTTTTCTCCAATTTCACGTAAAAATAAGCTTAACAATAACAATAAAAAAAGAAAGAAATAGTACATATTAGACCCATACTGACATGAGTCATTGCATTCTAGTATACACAGACAATATAATTTTAAATATTTCAAAAAATAATTAAATGACAATTTTGTTAAGTGTAAACTTTATTTTAGAAAAAGATCAAAAAAGAAGTAAAAAATATTTTGTAACAAAATTTCATATATTTAATAATTTTTTTTTAATTTACTCTTGATAACATATTTTCAGCTTAAATTTATTTTTTTAATGTATGTGTCTAATCAAACATCGGTGGCATAATTTTTTTTAGCATCAAAGGCACAGATCTGAAGATTTGGACGTTTGCAAATAATTAAACTATTTTTCAGTAAGGTGACAAGAAGTTAATAGTTTGATTTTAAAATGTTCAATGGATTCAAAAGTATCCCAAT

**GE1^76^** (SL4.0chr07, *Solanum* *pimpinellifolium* *cv* TS22)

Highlighted in green is the 76-bp insertion sequence.

AAATCAACATGTGCAAAATTCGTTTAAATTATTGTGAGGTGCTATAGATATGAAAAATCTTTATAACTAGAAAATTTAATAAGAATTGGGTCGGAATAATATTTTATTTATACTATTTTAGAATGATATTTTATTCATATTATTTTATTATTTTAAATATTTTTATCTTTTTAATTAAAATGGAAAAAGATTAATTTATTTTAAAATAAAATAAATATTATAATTTTTTAATTTTCTAATCTGATTAAATATTTTCATCGTTTAGCAATATACAAAAATATAATGTAGCTGTATTTTAAAAAGAAAAAAATCATTTTGACCAAAATTTAGCTAGAATGTATTTTATCCATATTATTTTTTACCTTTTTAAATATTTTTATTACTTTAACTTTCATGTTTTTAACTAAAAGTTCAAAAAAAATATTTTAATATAAAAGCTAGAATGATTTAATCGATATTATTTTTCAATATTTTTATTATTTTAACTTTCATATTGTTTAACTAAAAAAAAAAAAGATAAATTTATTTAATATAAAAAGAAATATAATAGACTTTTTAAAGTTTTGTTCTGGCTGGCCAGCCGTGGAACATAAATAAACGTGTGTAAAAATTCGTTGAAATTATTGTGAGGTGCTATAGATATGCACAGTTATTGTTATTAATAATTAATTATTGATGATCATAACCAACGACAAAAGGAAGTACATGTCATTTTTCTACATTTTGGTTTCTTTCTGGTAATTTAAATTTAGGAATGGGTTAAATAATTAGAAAATTTGGTTAGTATTTGGTTATGAAATTAAAATAATTATAATATTTTTTAATTTTAAAATAAATTGATTTTTATTTAATTTTTTATTTAAAAGATAAGAATAACAATATTTTAAAAGATAAAATAGCATAGAAAAATTATGATCTGATCAAAAAAAAAATTCCCTTTAAGTTAATAACTTCGATAATTAAATACTCTCACTATTTTTGGAGTTTTTTTATTATTTAAAATAATTAAATTAGTTAAGTTCATGATGAATATTTGATTTATTTATATTTATCCTTTTCTTTTTGATGAATTTTTATATCTTTTTAGAGATAAATTGCATTACTTTTAAAATTATATTTATAATTTTAAAATATAATTAAAATATTTACTTAACTATTTTCATTACAAATATATTGGATTATCTCACAAATGCAAATATATAGTATAAAAAGGACTGAAGGAGTGTTAACGAACGTACATCGGCCATAACACTTATGTAAACAAAAAATCAGAACAAAGTTACAACACTTACTTCGAAGTTCTATATATTTATATATTATATTAGAAAGGAGTTTCAAGTCTTTTTCAATATGTCAGTTTTCTGACATTAAATTAAAGTTTTGGTGGTATTAAGGCCAACTTCTAGAAATTAATATTCCATAAATTAATAATTTTTTTAAAATAATATTTTTTCTGATCATAATTTGGGCTAATAGAAAAAAAATCATCAATTTTGATGAGATAATATATTTCAGAAGACCCCTATATAAATATATTGTCCCATTGGTATCATAAATTAATAATTCTTTAAAGGTACAAATATACTTAAGATAATTTAGTGAAATGTGATTAGATTATGTTCTGTTTTTTCTTAAAATTTAAATCTAATTAAAGCCATCTCTAACTTTTCTTATTACATGGAGAGCTCCAATGTTGTCTCTTTAAACTACACCATAAATTTGTGAAGAGTTCTAGATACGATTAGGGTTCCTTATGTGGTTCCTCTAAACATGTATCACTTTCACCCGGATAATTCAACAGATTTTTGATATTGATTTTATTGTGGTAATCGAAATCATTAATCATAATCTCAAGTTAAACTCGTAGATAATTGAGAGTCTATAATTAAATTAATTAGTAGTGTATGCACATTTTCTTTATTTTTATCCTAATTGTTATTTTAACAATAACATATTAATAATAAATTTTAAAATATAATTAATAAAATTCTTCCGTAAAATGTTTTTGAAAAAAAAATTAAAAATAAGAACCTTGATGACTATTAAAATAAATGTATAATCTTTCGCTAATATATAAATAGTAATACTTTATATGTGCATGAAGTGTCGCTTTAAGTATGTAATGGTATAATATTTCCATGAGTAAAATAATTTATTATTTGAAAATCAACATGTTCATGGATCCTGAGCGTATTCAATTTTTCCTATGCAATACTATGCGTGATTAATAATTACTCAAGATGCGTTTGTTAATATTAAAAATATTACTTTTAGAAATAATTTCTAGGAAAATAAATATGTTTCTTTTTTCTTTTGATAGGTTGACAAAAAGTATTATCTTAAAAGTATTTTTATATAATTTAAATAAATATCATGTGTAAATTTTTTCATATGTCAATTATAAAAAATATTTTATTATTTTAAAAGAAAAAAATTATTTCCTAAAGAAAATATATTGTAAAAATTTTAACTGATTAAATATAGAAAAATTAACATTCTACATAATGAACACCACCTTAAAATTATTAAATTTCTTTTCTCCAATTTCACGTAAAAATAAGCTTAACAATAACAATAAAAAAAGAAAGAAATAGTACATATTAGACCCATACTGACATGAGTCATTGCATTCTAGTATACACAGACAATATAATTTTAAATATTTCAAAAAATAATTAAATGACAATTTTGTTAAGTGTAAACTTTATTTTAGAAAAAGATCAAAAAAGAAGTAAAAAATATTTTGTAACAAAATTTCATATATTTAATAAAAAAAATTTTTAATTTACTCTTGATAAAATATTTTCAGCTTAAATTTATTTTTTTAATGTATGTGTCTAATCAAACATCGGTGGCATAATTTTTTTTAGCATCAAAGGCACAGATCTGAAGATTTGGACGTTTGCAAATAATTAAACTATTTTTCAGTAAGGTGACAAGAAGTTAATAGTTTGATTTTAAAATGTTCAATGGATTCAAAAGTATCCCAAT

**>Solyc07g043480 *GAME17* promoter, 1621 bp**

GGTTGAGGATGAGATTATTGATAAGTGTTTTTTATGTTATGTTTATATGATCTTTTGATTATGAAATGTTCACATGACTTTATGATAATATGTCTTCGCTTATGATCATGCTTTATGTTAATGGAATCCTTAACTAGTATATGTTCTATGGTATGTGCATTGAAAAGGGAGAAAGCACCAACTCTTTTAAAGTCCAATTATATATATATATTTATATATATGAAATAAACATTTTAACTTCTTTTAAGTTAAAAATAATAACTATCTATTTTTTAAAAAAAAATAAAAATAAAAATCACACAACACATCCATTTGCGTCAAAGGAAAAGGTATGTAAAATTAAAAAAACAAGTAAATGTGAAAAGAGAAAATGTAAGGTAGTTCTCTAAAATTTCTAAAAGATAATTTAATTATGGGGGTGTTTTATCATATTAATACGAAATTTTAAAAAATAAATATCATCATACACTAATGACTGCATGACACGTGATAATTAATTTAAAATATAATTATATTTATCTTAAATTTTGCTTTATCCTTTTCTTTTTTATTTTTTCTCTTACTTTTTGACTAATTTTCAATTTTTTCTTTAATTTTAAATTTACCTTCCCACTTCACCCATCTCCCACGTCAGAACTGACCCGCTCCCCTCATTTTATTTCTTTTTTTCTTCAACTCCCATACTGCATATAGGTGTCACTCTCTCTACATGTGAACAAACCACACAAGACGCAACATGAATTTAACTCCTTTTCATTATTTATTCTTTTCATTTTTCAAGTATATATTTGGATGAAAATCATATCTTATAATTAAAAATTTATTTATTATTTTAAATCATCACACATGACTTCTATTTAATTAATAAAAAGATATGTATTCACTCAACACATCCATTCGCCCCCCACACCCCACTCCCAGCACCTATTTAACATACAGTACACCCATTATGCGCACGTGAAAAGGTAGTAAATTCTTCTTCTTCTTCTATATAATAAAAAATAAAAACTAAATTTTTAAAATTTTATTTGTGTCATATAAATTGAAACAGTGAAAATAATGTATATTATTAGTGACAATAATTAATGATAAACGACATTTGATTGAATTTTAAATTTCACACGTGATACATAGATTGAGATTATTATTTGACAATTAAAATTTAAATTAACAAATTACAATAACTGACAACTAAATTTTTATTTTTATTAAAAGTTTGATTGAATTTCAAAATTTCATGTGTCGAATAAATTGAGATAGAAATAGTTATATATATATATGTATATCAATTGAAAACTATTTTAGAAGTATTATAAATCATAATGATTAATATATAATTTAAAGTAGTTAAAATTTAACATGATTCAAAATACATAAATTAATGGACTTATTTATGATATTTTCTTCTCAACACATTGAAGTATATATTTGGAAAAATGATGAATCTAAATTTTAGGCTGTCTGTTTAAATTATTATTTTCATATGCAAACATTAAAAATACGGTTGTTGGTATTCGGTAGTTGTTACTTCATATCTATATATCCTTGTTTAGAGTAACACTCCAATAAGTCTTCAAAAAAAAAAAAAAATACTAATAGTTATACTCCACAACATCTTCAC

**>Solyc07g043490 *GAME1* promoter, 1565 bp**

GTAAGTATGATCAAGAACGGAGTGAGTAAGGTTTAGAACCTCTAAAGCATTTGGGAATTATAAAGGTAGAGGAATGAACAATCTTATTGCATGTTTCTTACTAGTCGGTTTCTTATATTAAGTGGTGGGCATCAAATTGACAGATGATACCTACCAGTATGTATGGTTGTACTGATACTACCCTTGCTTTACCTTTTTTTGACATAGTTTGGTTGCAGTATTAGTGATGTGACATAGATAAAGACGAAGTCAATCTCTAATAGCTTCTATTTCTCCTTCCGCATGGTGGGAGCTGCGTCATTTATAATTTTGTTATATTTGTACTCTTAGTAGCTCTTGTACCTATTTGGGCTAGATCCCTGAGATGTTAATTACTTTACAAGTCTTAACTTTGAGTCATTTCAAAACTCTTATTTTATAAAACGTTACTTGAATTTCTTTCAGTAGTTAGTTTTCTTAATTTTCGCATTTGTTCAAAAGTTTAGGAATTGAGGGTTCTCTTACTGAAGTGTAGACCGCACGGTCCTGTGGGTTGAATCGTGACACTTAGACTAAAACACAACTAAGGAGATTGCTTAAAGAGAGAATACCACGTGATGTCCCCCACACCACACCTGCTAAGAAACGAGATCTCAATGTTAAAACTTATAGAGATAATGCACAAGTACCCCTTCAATGTATGCGCGAAATCTCAGAGACACACTTATACTATTCTAAGGTCCTATTATTCCCCTAAACTTATTTTATTAATAATTCTCTACCCCTTTTTGACCTACTTGACACTATTTTGTGGGTCCAACGCTGATTAACTATTTTTCAAGCTAATACTATGTAGGCCGAAAAGGGGTAGAAAATTAGTTAGAAAATAAGTTCAGGGGGGTAATAGGACCTTAGTATAGCATAAATGTGTCTCTGGAATTTCGGGTATCGGTTGAGGGGGTACTTGTGCATTTTTCCAAACTTATATGATGAACTTGTAATGCACATAGAGGGAAGTAGAAGCTCGATATGAACCTTACGACTCAGACTCCTTTCCCCTACGATGAGAATCGCGTTGCAACCCTTTGTTTGGTGGATTTGGGAAGGAAGTTGAAATGAGATTGCAATTTATAATATTTTCCATATAATTTTTGAATATCAAAATTTTTTACTTAACATATTGAATCAATGTAATCTAATTTAAATTTTAAAATTAATCAAATTAACTTTTGAAAAATATTTCCGGAGGGGTGGATCAGAAAGAAAAGGTCTTAGTTTCACAAAGGATACCAAAGATTAATGTCATTTAATTGGATTTAAAATACTTCATGTGTCTCTTTATTTTTAAATTGACACACTTATTAAGAAAACAATTATTATCATAGTGAATTTACCATTTTACTCATATTCTTACGAAGTGAATGGATTAAAAAATTAAGATTTTTTTGTAAATTCAAATGATTAATTAAAGATATATAATCAAGGAAGATTAAACTCGCCATTCATTAGTCTATAAATATAAAGATGAGTAACAGAGCAAGGTATAAGAGTTGTTAAACAACAACTGTTCTTTGTATTGGGGTAGT

**>Solyc07g043500 *GAME18* promoter, 1585 bp**

ACAGTAAAAAACCCTCTTAATGGGTCTTGAGATATCTGAATAAATCATATCTATAGGAGACTCTTAATAACATTTAGACTCATTTGATAAGTTATTAAATAATAAATCATAGTTTGTTTGCTTTGAGTATGCATTTTTATCTGATAATTAAAAACTTTTTTTCACTTTTATCAAGTATACACTATATTTTTCCCAACTGGTAAATTTTCATAAATCCTTTCAAATATTAATAACTTTCTTGTATTGATGTGTGTCAAGAACACTGCTTGCAATAATATTTTTGGTATATTGTTATTTATCAAGGTTTTTCAATGAAAAAAATAATACTCCAAAACATAATTATTAAAACTTTTAAAACTTTTTTTTTTATCAAAAGTGATATACTTTGTTAAAATGAAAATTTTATAATATTTTATCAATATAATGTTTGATTTCAATTCCACATTTAGATTCAGATATTGAAAACAAACAACATTAATTACTTACTGTTCAGATTTAGAGATCAGATTTTCATATTCAGATCTGTATTCAAATCAAAACATCCAGATCTTAATGCAAATCTTAATATTCAGATGTGTATTCAGATTCAGATCTCTTAATCTTAACGAAAACAAATGAGGCCTAAAACCCACTCATCTAATAAGAGATGGCGTTCAATATTCAGTTCAGGTTTTTTTTTTAATTTCAGATTTGGTAATTTCAATAATTGATAATAGAAAATAGAAATTAAGTATTTTTTTTAAAAAAAATGTTTAAATAATTCAATGAACAATAAATTTAATTTAATTTGATAGTATTCAGTAATACAGTACTGAAGTTATGGCATTGTATTCCTAATACTATTATTTTTCCTATTATTTTCATTTTTTATGTGGAGACATGATAATATAGAAGATCAACAAGTAAGAATACATTAAGAAAAGTAAATTGATACAACTAAAAGACATACGTTTAAAAAAGCAAATTGATCAAGTCGTTTGGTAGAGTGTATAAAAAATATTTATTTATGCATTAGTCTTGTATATTACTAGTACCTGATTTGGTTCTTTTTTTGCAACTTAGTACAGTTGATTTCTAGGTATTAGCAATGCAATATTTTTAATGCATGCATTAACTCAGTTAAAAAACCAATTGCCCCTAATACCTTTTTATATTTTTTCGCCATAATAGTGAAGCGTATCGAAAAATACATTTATGTAAATTTAATGCAAACTTAACTATTACACTCTATTTTGCATTATATTAATACACTCTACCAAATAACTATAACTTTCTTTTAAACTTATATTAATGAATAATAAATTTGATAATTTAATTTTCGATATTCTATGTCCAGTCCTACTTCTAATATATTAGGGGGGGAAAATATTGCTTGGACTTGGCTCACCTACCTTCAGTAACATATCTCTCAGGCTTTGATAGGTTTAGACATGTGTAATTATTTATTTATTTAATGGTTAAGATAAAAATTCATTTGGTCAATCAAGGAAATTTAACTATGGTTAATGGTTACTGAGGTGGATCCAATTTGTTGTTCTATAAAGAGCTTCTTCTTCATTGAGAAAAAAATACAGAGACACAAGTG

**>Solyc07g043500 *GAME18* - CDS, 1326 bp**

ATGGGAAATATGAGAATTGTAATGTTGCCATACTTAGCTTATGGCCACATCACACCTTTTCTTGAACTAGCCAAGAAACTCTCAAACAGAGGTTTCTCCATTCACATATGTTCTTCTCCCATCAATCTTAGCTTCATCAAGGCAAAAATCCCAGAAAAGTACTCTTCCTCAATTCATTTAGTTGAACTTCACTTACCAAATTTACCTGAACTTCCTCCTCATCACCACACTACCAATGGCCTTCCAAATCATCTTAAACAAACTCTTTTCAAAACACTTAAGATGACCAAACCACAGCTTCACCAAATCTTGAGTGACTTGAAACCTGATTTTTTTATTTATGATATCATGTTACTATGGTCAGCTGTAGTTGCATCTTCACTCAACATCCCATCGTTAAGATTCTACACTGTAAACGCAGCCATTTTTAGTTATTTTTTCCATTTTTATTTCAATCCAGGAGAGGAATTCCCTTTCCCAGCTCTCTATATGAGGGATTATGAGCTAGCGAAGATGACACATGAAGTTGCTGATGATGCGGAGGTTGAAGTTGACAGAGACAAAGTTACTGAGTCCGATAAATTTGTACTTGTTCATAGCACTAAATCAATAGATGGGAAGTACATGGATTATCTTTGCGGAACAGGGCAGGCAAAAGTTGTACCAATTGGAACAGAATCTCCTGAAGATGGTGTTGGTGATGTCGATAAGATTGATATCGAACTTGTCAAATGGCTTGAGAAGAAAACAGAGCATTCAACTGTTTACGTTTCATTTGGGAGTGAGTATTTCTTAAGCAAAGAAGAAATGGAAGAGGTAGCCTATGGATTAGAGGTTAGCGGTGTAGATTTCATATGGGTTGTTAGGTATCAAAAAGGGGAACAATTAGAACTTCCACAAGGTTTTAAAGAAAGAATTGGAGATAGAGGAAGGATCATTGAAGGATGGGCTCCGCAACAAAGAATTCTAAAGCATTCAAGTATTGGTGGATTTGTAACGCATTGTGGTTGGAATTCGACGTTAGAGAGCATAGAATTTGGTGTTCCAATCATAGCTATGCCTATGCTTTATGATCAGCCTTTGAATGCTAGATTGATGGTCGAAAATGGAGTAGCTGTTGAGGTCCCAAGAGATGAAAAAGGGAATCTTGATAGAGTAAATATAGCTGAGAAAATTAAACATGTGATTCGTGACGAAACAGGGGAGAATTTGAGGAAGAAGATGAATAATCTTGGTGAGAATGTGAGATCTCAAAGAGAAGAGGAGATGGATGGAGTTGTTAAGGTTATACAACTCTTGATTGATGAGAAGAAAGGGACCTTATGA

**>Solyc07g0452700 *AGL66* - CDS, 573 bp**

ATGAAGAAAATTGAGGATTCAACATCCCGTAAACAGTTCTATTCAAATCGCAAGGATAGCATTGTGAAGAAGTCAAATGAGCTGGCGGTTGTATGTGGTACAGATGTGGGGTTGTTGATGTTTTCTCCATCTGGTCAGCTGACTACCTATTCTAGCAAAGAAAGTATTGAGGACATCATGATCGAAGCTATGAACAAGTCTGTGAATCCGCGACCCATACCAAATCTAAATGAACAGCTTTTGATGCAGAGTCTCAAACAGTCAAAATCTGAAGGCCAAATGGTTGGAAAAATAGCTATTGCTGAGGCTCATGAGAAGAAGCTTAATGAGCTCAAAGAAACACTAAGGGAGGCACAACAGAAAATAAGGTATTGCAATCCGCAAGTGGAGAATATCAGCTCAGTCCAAGAAGCTGAAGCATATGAGCAGTTCCTTAGGAGTAATATGGAACAGATTCAACAATCAAAAGCAAAACTCTTAGGTGTCCAAGGATTAGTCCATAGAAATGAATATCCTGCGGTCAACACAGAGGATACGGCTGCTGCAGGAACCAGTAGTGGATGGATGTTTTGA

**>Solyc08g076930 *MYC2* - CDS, 2070 bp**

ATGACTGAATACAGCTTGCCCACCATGAATTTGTGGAACAATAGTACTAGCGATGATAACGTTTCTATGATGGAAGCTTTTATGTCTTCTGATCTTTCTTTTTGGGCTACTAATAATTCTACTTCTGCTGCTGTGGTTGGTGTCAATTCAAATCTTCCTCATGCTAGTAGTAATACTCCCTCTGTTTTTGCACCATCTTCTTCTACATCTGCATCTACTTTATCCGCAGCTGCGACTGTGGATGCTTCCAAATCTATGCCGTTTTTCAACCAAGAAACCCTTCAGCAGCGTCTTCAAGCTCTTATTGATGGTGCTAGAGAGACGTGGACTTATGCTATCTTTTGGCAATCGTCGGTTGTTGATTTCTCAAGTCCGTCTGTGTTGGGTTGGGGAGATGGTTATTACAAAGGGGAAGAAGATAAAGCAAAAAGGAAATTATCGGTGTCATCACCTGCTTATATTGCTGAGCAGGAGCATCGGAAGAAGGTTCTACGGGAGCTGAATTCGTTGATTTCCGGGGCACCACCCGGAACGGATGATGCGGTTGATGAAGAAGTTACCGACACCGAATGGTTCTTTCTTATCTCCATGACCCAATCGTTTGTTAATGGAAGTGGGCTTCCTGGTCAGGCGTTGTATAGTTCCAGCCCGATTTGGGTCGCCGGAACTGAGAAATTGGCAGCTTCACACTGTGAACGTGTGAGGCAAGCACAAGGGTTCGGGCTTCAGACGATTGTCTGTATTCCTTCAGCTAACGGCGTGGTTGAATTGGGCTCGACGGAGTTGATTGTTCAAAGTTCTGATCTTATGAACAAGGTTAGAGTATTGTTTAACTTCAGTAATGATTTGGGTTCTGGTTCATGGGCTGTGCAGCCGGAGAGCGACCCATCGGCGCTCTGGCTCACTGATCCATCGTCCTCAGGTATGGAAGTTAGAGAGTCTTTAAATACAGTTCAAACAAATTCAGTTCCATCTAGTAATAGTAATAAGCAAATTGCTTATGGAAATGAGAATAATCATCCATCTGGAAATGGTCAGAGTTGTTACAATCAGCAACAACAGAAGAATCCTCCTCAGCAACAAACACAAGGATTCTTCACGAGGGAGTTGAATTTTTCGGAATTCGGTTTCGATGGAAGTAGTAATAGGAATGGAAATTCATCGGTTTCTTGCAAGCCTGAATCAGGAGAAATCTTGAATTTTGGTGATAGTACTAAAAAAAGTGCTTCCAGTGCCAATGTGAACTTGTTTACAGGTCAGTCCCAATTTGGGGCTGGGGAGGAGAATAATAACAAGAACAAGAAAAGATCAGCTACTTCCAGGGGAAGCAATGAAGAAGGAATGCTTTCATTTGTTTCAGGTACAGTTTTGCCTTCTTCGGGCATGAAGTCAGGTGGAGGCGGAGGCGAAGACTCTGAACATTCAGATCTCGAGGCTTCAGTGGTGAAAGAAGCTGATAGTAGTAGAGTGGTAGAGCCTGAAAAGAGGCCAAGGAAGCGAGGTAGAAAGCCAGCGAATGGACGGGAGGAGCCATTGAATCACGTCGAGGCAGAGAGGCAAAGGAGGGAGAAATTGAACCAAAGATTCTACGCGCTTAGAGCTGTTGTACCAAATGTGTCTAAGATGGACAAGGCATCACTCCTTGGAGATGCTATTTCCTATATAAACGAGTTGAAATCGAAGCTTCAAAATACAGAGTCAGATAAAGAAGACTTGAAGAGCCAAATAGAAGATTTAAAGAAAGAATCAAGGCGCCCCGGTCCTCCTCCACCACCAAATCAAGATCTCAAGATGTCTAGCCACACTGGAGGCAAGATTGTAGACGTGGATATAGACGTTAAGATCATCGGATGGGATGCAATGATTCGTATACAATGTAATAAAAAGAATCATCCAGCCGCAAGGCTAATGGCAGCGCTCATGGAATTAGACCTAGACGTGCATCATGCCAGTGTTTCAGTTGTCAACGATTTGATGATCCAACAAGCCACAGTGAAAATGGGTAGCAGACATTACACTGAAGAGCAGCTTAGGGTAGCGTTGACATCGAAAATTGCTGAAACACACTAA

**>Solyc01g090340 *GAME9* - CDS, 678 bp**

TATTCATCCAAAACAAGAATGAGTATTGTAATTGATGATGATGAAATCTTCTCTTTACCTAGCCTTGATGAACTTGAATCCATCACACATCTTCTTTATGACGACGATTCCGATTTTTTCGAAACTCTTTCCCCAATGAGTTTAGATGTTACAACATTATTGCCTAATATTCCTACCTCCAATTCAATTGAATCCCCCGTAACACCGGAGGAAACAAAAGAACCATCTGTGGCGTGTGAGGACGCGCCACAAGATTGGAGGCGGTTCATAGGGGTGAGGCGGAGGCAGTGGGGCACGTTTTCAGCCGAAATAAGAGATCCAAATAGGAGAGGAGCGAGGCTGTGGCTCGGAACTTATGAGTCCCCGAGGGATGCAGCATTAGCTTATGACCAAGCCGCTTACAAGATTCGGGGAACCAAAGTTCGGCTTAATTTTCCTGACCTGATTGGCTCGGACGTACCTATGCCACCTAGAGTAACGGCTAGGCGTCGTACACGCTCACGCTCACGCTCACCCGAGCCATTAACAACTTCGTCCTCGTCATCCTCATCATCCTCGTCCTCGTCCTCGTCCTCGTCGGAAAATGGAACGAAGAAAAGGAAAATAGATTTGATAAACTCAATAGCAAAATCCAAATTACTTTGTGGGATGGATTTACAAATGTTAATACAAATGTGA

**> Solyc01g090340 *GAME9* - Protein, 689 AA**

Highlighted in yellow is the 135th amino acid.

MSIVIDDDEIFSLPSLDELESITHLLYDDDSDFFETLSPMSLDVTTLLPNIPTSNSIESPVTPEETKEPSVACEDAPQDWRRFIGVRRRQWGTFSAEIRDPNRRGARLWLGTYESPRDAALAYDQAAYKIRGTKVRLNFPDLIGSDVPMPPRVTARRRTRSRSRSPEPLTTSSSSSSSSSSSSSSSSENGTKKRKIDLINSIAKSKLLCGMDLQMLIQM*
