# Supplementary material for: A distal enhancer guides the negative selection of toxic glycoalkaloids during tomato domestication
Source: Nat Commun. 2024 Apr 3;15:2894. doi: 10.1038/s41467-024-47292-7 (PMC10991328; doi:10.1038/s41467-024-47292-7)
Supplement: Supplementary file 16 — Reporting Summary [file 41467_2024_47292_MOESM16_ESM.pdf]

nature portfolio

Corresponding author(s): Mingchun Liu

Last updated by author(s): Mar 6, 2024

Reporting Summary

Nature Portfolio wishes to improve the reproducibility of the work that we publish. This form provides structure for consistency and transparency in reporting. For further information on Nature Portfolio policies, see our Editorial Policies and the Editorial Policy Checklist.

Statistics

For all statistical analyses, confirm that the following items are present in the figure legend, table legend, main text, or Methods section.

n/a	Confirmed	
☐	☒	The exact sample size (*n*) for each experimental group/condition, given as a discrete number and unit of measurement
☐	☒	A statement on whether measurements were taken from distinct samples or whether the same sample was measured repeatedly
☐	☒	The statistical test(s) used AND whether they are one- or two-sided<br>*Only common tests should be described solely by name; describe more complex techniques in the Methods section.*
☐	☒	A description of all covariates tested
☐	☒	A description of any assumptions or corrections, such as tests of normality and adjustment for multiple comparisons
☐	☒	A full description of the statistical parameters including central tendency (e.g. means) or other basic estimates (e.g. regression coefficient) AND variation (e.g. standard deviation) or associated estimates of uncertainty (e.g. confidence intervals)
☐	☒	For null hypothesis testing, the test statistic (e.g. *F*, *t*, *r*) with confidence intervals, effect sizes, degrees of freedom and *P* value noted<br>*Give P values as exact values whenever suitable.*
☐	☒	For Bayesian analysis, information on the choice of priors and Markov chain Monte Carlo settings
☐	☒	For hierarchical and complex designs, identification of the appropriate level for tests and full reporting of outcomes
☒	☐	Estimates of effect sizes (e.g. Cohen's *d*, Pearson's *r*), indicating how they were calculated

Our web collection on statistics for biologists contains articles on many of the points above.

Software and code

Policy information about availability of computer code

Data collection	SCIEX OS (v1.7);Bio-Rad CFX manager (v3.1)
Data analysis	GraphPad (v8.02);FastQC(v0.11.9), fastp (v0.22.0), BWA (v0.7.17), HISAT2 (v2.2.1), Samtools (v1.13), Juicer (v0.6.15), Hiccups (v0.8.9), HiCExplore (v3.7.2)

For manuscripts utilizing custom algorithms or software that are central to the research but not yet described in published literature, software must be made available to editors and reviewers. We strongly encourage code deposition in a community repository (e.g. GitHub). See the Nature Portfolio guidelines for submitting code & software for further information.

Data

Policy information about availability of data

All manuscripts must include a data availability statement. This statement should provide the following information, where applicable:

- Accession codes, unique identifiers, or web links for publicly available datasets
- A description of any restrictions on data availability
- For clinical datasets or third party data, please ensure that the statement adheres to our policy

The whole genome resequencing data, the RNA-Seq data and the Hi-C sequencing data have been deposited in the Genome Sequence Archive (GSA) under accession number CRA011801. The DNase-seq data, and ChIP-seq data (H3K27ac, H3K4me3, H3K27me3 and MYC2) were downloaded from NCBI (BioProjects: PRJNA381300, SRA046131 and PRJNA375842)

Research involving human participants, their data, or biological material

Policy information about studies with human participants or human data. See also policy information about sex, gender (identity/presentation), and sexual orientation and race, ethnicity and racism.

Reporting on sex and gender	N/A
Reporting on race, ethnicity, or other socially relevant groupings	N/A
Population characteristics	N/A
Recruitment	N/A
Ethics oversight	N/A

Note that full information on the approval of the study protocol must also be provided in the manuscript.

Field-specific reporting

Please select the one below that is the best fit for your research. If you are not sure, read the appropriate sections before making your selection.

☒ Life sciences ☐ Behavioural & social sciences ☐ Ecological, evolutionary & environmental sciences

For a reference copy of the document with all sections, see nature.com/documents/nr-reporting-summary-flat.pdf

Life sciences study design

All studies must disclose on these points even when the disclosure is negative.

Sample size	A minimum sample size of 3 independent biological experiments was chosen to obtain valid statistical analyses. Sample size has been described in each individual experiment.
Data exclusions	No data were excluded from the analyses.
Replication	All replication were successful performed independently. Number of replication has been describe in each individual experiment
Randomization	The UPLC-MS/MS analysis orders were randomized.
Blinding	No investigations were blinded because blinding was not seriously relevant to our plant experiments (non-clinical trials

Reporting for specific materials, systems and methods

We require information from authors about some types of materials, experimental systems and methods used in many studies. Here, indicate whether each material, system or method listed is relevant to your study. If you are not sure if a list item applies to your research, read the appropriate section before selecting a response.

Materials & experimental systems

n/a	Involved in the study
☐	☒ Antibodies
☒	☐ Eukaryotic cell lines
☒	☐ Palaeontology and archaeology
☒	☐ Animals and other organisms
☒	☐ Clinical data
☒	☐ Dual use research of concern
☒	☐ Plants

Methods

n/a	Involved in the study
☒	☐ ChIP-seq
☒	☐ Flow cytometry
☒	☐ MRI-based neuroimaging

Antibodies

Antibodies used	anti-H3K27ac (Cell Signaling Technologies; #8173), anti-RNA polymerase II (Abcam; #ab 264350), anti-FLAG(Cell Signaling Technologies; #14793), anti-HIS (Cell Signaling Technologies; #9991), anti-GST (Cell Signaling Technologies; #2622).

Validation

The antibodies, including (anti-H3K27ac, anti-RNA polymerase II, anti-FLAG), were used for ChIP-qPCR. Anti-HIS and anti-GST were used for DNA pull-down. Anti-H3K27ac: For optimal ChIP and ChIP-seq results, use 5 µl of antibody and 10 µg of chromatin (approximately 4 x 106 cells) per IP. This antibody has been validated using SimpleChIP Enzymatic Chromatin IP Kits. Western blot analysis of extracts from HeLa and C2C12 cells, untreated (-) or treated (+) with Trichostatin A (TSA) #9950 (1 µM, 18 hr), using Acetyl-Histone H3 (Lys27) (D5E4) XP® Rabbit mAb (upper) or Histone H3 (D1H2) XP Rabbit mAb #4499 (lower). (https://www.cellsignal.cn/products/primary-antibodies/acetyl-histone-h3-lys27-d5e4-xp-174-rabbit-mab/8173).

Anti-RNA polymerase II: RNA polymerase II RPB1 was immunoprecipitated from HEK-293T (human epithelial cell line from embryonic kidney transformed with large T antigen) whole cell lysate (0.5 or 1.0 mg per IP reaction; 20% of IP loaded) using ab264350 at 6 µg per reaction. Western blot was performed on the immunoprecipitates using an alternative RNA polymerase II antibody (https://www.abcam.cn/products/primary-antibodies/rna-polymerase-ii-rpb1-antibody-ab264350.html).

Anti-flag: Western blot analysis of extracts from 293T cells, mock transfected (-) or transfected with DYKDDDDK-GFP (N-terminal DDK-Tag; +), GFP-DYKDDDDK (C-terminal DDK-Tag; +), human CASQ1-DYKDDDDK (C-terminal DDK-Tag; +), or human FoxG1-DYKDDDDK (C-terminal DDK-Tag; +) as indicated, using DYKDDDDK Tag (D6W5B) Rabbit mAb. For optimal ChIP results, use 10 µl of antibody and 10 µg of chromatin (approximately 4 x 106 cells) per IP. This antibody has been validated using SimpleChIP Enzymatic Chromatin IP Kits. (https://www.cellsignal.cn/products/primary-antibodies/dykddddk-tag-d6w5b-rabbit-mab-binds-to-same-epitope-as-sigma-aldrich-anti-flag-m2-antibody/14793).

This Cell Signaling Technology antibody is conjugated to the carbohydrate groups of horseradish peroxidase (HRP) via its amine groups. The HRP conjugated antibody is expected to exhibit the same species cross-reactivity as the unconjugated His-Tag (27E8) Mouse mAb #2366. Western blot analysis of extracts from COS-7 cells, mock-transfected (-) or transfected with a construct expressing C-terminal His-tagged Tyro3 (His-Tyro3; +) using His-Tag (27E8) Mouse mAb (HRP Conjugate) (https://www.cellsignal.cn/products/antibody-conjugates/his-tag-27e8-mouse-mab-hrp-conjugate/9991).
Western blot analysis of extracts from untransfected control cells (-) and transfected cells overexpressing GST-Bad (+), using GST-Tag Antibody (left) and Bad Antibody #9292 (right) (https://www.cellsignal.cn/products/primary-antibodies/gst-tag-antibody/2622).
